# The Tree of Sex consortium: a global initiative for studying the evolution of reproduction in eukaryotes

**DOI:** 10.1093/jeb/voaf053

**Published:** 2025-05-08

**Authors:** Daniel Jeffries, Chiara Benvenuto, Astrid Böhne, Christelle Fraïsse, Sònia Garcia, Paul Jay, Lukáš Kratochvíl, Caitlin E McDonough-Goldstein, Aurora Ruiz-Herrera, Cibele G Sotero-Caio, Nicole Valenzuela, Melissa A Wilson, Tree of Sex Consortium, Aleksandra Bliznina, Valentina Peona, Thomas Desvignes, Aparna Lajmi, Christina N Hodson, Yann Guiguen, Mónica Moura, Paul Jay, Tanja Slotte, Bjarte H Jordal, Michael A White, Philipp Schiffer, Maja Lazarević, Christopher G Wilson, Joana Isabel Meier, Joris M Koene, Christelle Fraisse, Mathias Scharmann, Bahadir Onsoy, Jun Kitano, Giulia Lin, Ann Kathrin Huylmans, Emmarie P Alexander, Caitlin E McDonough-Goldstein, Sara Calhim, Ashwini V Mohan, Shana Pau, Sophie Helen Smith, Cecile Molinier, Melissah Rowe, Leo W Beukeboom, Stephen I Wright, Matjaž Kuntner, John R Pannell, Ludovic Dutoit, Edina Nemesházi, Tony Gamble, Peter Szoevenyi, Petra Bulankova, Vratislav Peska, Caleb J Krueger, Astrid Böhne, Dragana Cvetković, Mércia Patrícia Pereira Silva, Thaís Elias Almeida, Frédéric Veyrunes, Agnieszka Lipinska, Veronika Bókony, Jana C Vamosi, Peter Glasnović, J Antonio Baeza, Melissa A Wilson, Tatiana Giraud, Cibele G Sotero-Caio, Stuart V Nielsen, Sònia Garcia, Richard Challis, Lukáš Kratochvíl, Luohao Xu, Marta Turon, Jie Wang, Deborah Charlesworth, Charlotte J Wright, Bozena Kolano, Brendan J Pinto, Giacomo Potente, Wagner Luiz dos Santos, Zoran Marčić, Clare E Holleley, Soleil E Young, Stuart F McDaniel, Lucija Andjel, Matthew W Hahn, Claudia C Weber, Fredric J Janzen, Lewis Stevens, Ahammad Kabir, Jennifer D Gresham, Paul A Saunders, Marine Arakelyan, Jiří Král, Duminda S B Dissanayake, Stacy A Krueger-Hadfield, Qi Zhou, Jonathan M D Wood, Catherine L Peichel, Karel Janko, Maria Immacolata Ferrante, Chiara Benvenuto, Michail Rovatsos, Andrew J Mongue, Máire Ní Leathlobhair, Jessica K Abbott, Jonna Kulmuni, Anna Torgasheva, Alexander Suh, David H Lunt, Susana M Coelho, Sam Ebdon, Alex Makunin, Melissa A Toups, Jens Bast, Quentin Helleu, Quentin Helleu, Tony Heitkam, Vladimir Trifonov, Sadye Paez, Rainer Melzer, Natalia Borowska-Zuchowska, Nicole Valenzuela, Daniela H Palmer Droguett, Rita Monteiro, Petr Nguyen, Kamil S Jaron, Daniel L Jeffries, Caroline Howard, Tanja Schwander, Kerstin Howe, Ravinder K Kanda, Arthur Georges, Wen-Juan Ma, Ore Francis, Mark Blaxter, Sarah P Otto, Roman Hobza, Louise D Heitzmann, Vasiliki Kousteni, Aurora Ruiz-Herrera, David Peris, James Umen, Cristiana Ramalho Maciel, Alexandr Sember, Ricardo Utsunomia, Yukako Katsura, Matthias Stöck, Simon Kershenbaum, Tymoteusz Pieszko, Vilma Loreto, Martha Mercy Mulongo, Kamil S Jaron

**Affiliations:** Division of Evolutionary Ecology, Institute of Ecology and Evolution, University of Bern, Bern, Switzerland; Environmental Research and Innovation Centre (ERIC), School of Science, Engineering and Environment, University of Salford, Salford, United Kingdom; Centre for Molecular Biodiversity Research, Leibniz Institute for the Analysis of Biodiversity Change LIB, Museum Koenig Bonn, Bonn, Germany; CNRS, Univ. Lille, UMR 8198 – Evo-Eco-Paleo, Lille, France; Institut Botànic de Barcelona, IBB (CSIC-CMCNB), Barcelona, Spain; Center for GeoGenetics, University of Copenhagen, Copenhagen, Denmark; Department of Ecology, Faculty of Science, Charles University, Prague, Czech Republic; Department of Integrative Biology, University of Wisconsin-Madison, Wisconsin, United States of America; Departament de Biologia Cel·lular, Fisiologia i Immunologia, Universitat Autònoma de Barcelona, Cerdanyola del Vallès, Spain; Genome Integrity and Instability Group, Institut de Biotecnologia i Biomedicina, Universitat Autònoma de Barcelona, Cerdanyola del Vallès, Spain; Tree of Life, Wellcome Sanger Institute, Wellcome Genome Campus, Cambridge, United Kingdom; Department of Ecology, Evolution, and Organismal Biology, Iowa State University, Ames, IA, United States; School of Life Sciences, Arizona State University, Tempe, AZ, United States; Center for Evolution and Medicine, Arizona State University, Tempe, AZ, United States; Center for Mechanisms of Evolution, Biodesign Institute, Tempe, AZ, United States; Tree of Life, Wellcome Sanger Institute, Wellcome Genome Campus, Cambridge, United Kingdom

**Keywords:** biodiversity, asexual reproduction, sexual reproduction, sex chromosomes, sex determination, database, ontology

## Abstract

Reproduction is a fundamental aspect of life that affects all levels of biology, from genomes and development to population dynamics and diversification. The first Tree of Sex database synthesized a vast diversity of reproductive strategies and their intriguing distribution throughout eukaryotes. A decade on, we are reviving this initiative and greatly expanding its scope to provide the most comprehensive integration of knowledge on eukaryotic reproduction to date. In this perspective, we first identify important gaps in our current knowledge of reproductive strategies across eukaryotes. We then highlight a selection of questions that will benefit most from this new Tree of Sex project, including those related to the evolution of sex, modes of sex determination, sex chromosomes, and the consequences of various reproductive strategies. Finally, we outline our vision for the new Tree of Sex database and the consortium that will create it (treeofsex.org). The new database will cover all Eukaryota and include a wide selection of biological traits. It will also incorporate genomic data types that were scarce or non-existent at the time of the first Tree of Sex initiative. The new database will be publicly accessible, stable, and self-sustaining, thus greatly improving the accessibility of reproductive knowledge to researchers across disciplines for years to come. Lastly, the consortium will persist after the database is created to serve as a collaborative framework for research, prioritizing ethical standards in the collection, use, and sharing of reproductive data. The new Tree of Sex consortium is open, and we encourage all who are interested in this topic to join us.

**Tree of Sex Consortium:**Aleksandra Bliznina, Valentina Peona, Thomas Desvignes, Aparna Lajmi, Christina N. Hodson, Yann Guiguen, Mónica Moura, Paul Jay, Tanja Slotte, Bjarte H. Jordal, Michael A. White, Philipp Schiffer, Maja Lazarević, Christopher G. Wilson, Joana Isabel Meier, Joris M. Koene, Christelle Fraisse, Mathias Scharmann, Bahadir Onsoy, Jun Kitano, Giulia Lin, Ann Kathrin Huylmans, Emmarie P. Alexander, Caitlin E. McDonough-Goldstein, Sara Calhim, Ashwini V. Mohan, Shana Pau, Sophie Helen Smith, Cecile Molinier, Melissah Rowe, Leo W. Beukeboom, Stephen I. Wright, Matjaž Kuntner, John R. Pannell, Ludovic Dutoit, Edina Nemesházi, Tony Gamble, Peter Szoevenyi, Petra Bulankova, Vratislav Peska, Caleb J. Krueger, Astrid Böhne, Dragana Cvetković, Mércia Patrícia Pereira Silva, Thaís Elias Almeida, Frédéric Veyrunes, Agnieszka Lipinska, Veronika Bókony, Jana C. Vamosi, Peter Glasnović, J. Antonio Baeza, Melissa A. Wilson, Tatiana Giraud, Cibele G. Sotero-Caio, Stuart V. Nielsen, Sònia Garcia, Richard Challis, Lukáš Kratochvíl, Luohao Xu, Marta Turon, Jie Wang, Deborah Charlesworth, Charlotte J. Wright, Bozena Kolano, Brendan J. Pinto, Giacomo Potente, Wagner Luiz dos Santos, Zoran Marčić, Clare E. Holleley, Soleil E. Young, Stuart F. McDaniel, Lucija Andjel, Matthew W. Hahn, Claudia C. Weber, Fredric J. Janzen, Lewis Stevens, Ahammad Kabir, Jennifer D. Gresham, Paul A. Saunders, Marine Arakelyan, Jiří Král, Duminda S.B. Dissanayake, Stacy A. Krueger-Hadfield, Qi Zhou, Jonathan M.D. Wood, Catherine L. Peichel, Karel Janko, Maria Immacolata Ferrante, Chiara Benvenuto, Michail Rovatsos, Andrew J. Mongue, Máire Ní Leathlobhair, Jessica K. Abbott, Jonna Kulmuni, Anna Torgasheva, Alexander Suh, David H. Lunt, Susana M. Coelho, Sam Ebdon, Alex Makunin, Melissa A. Toups, Jens Bast, Quentin Helleu, Quentin Helleu, Tony Heitkam, Vladimir Trifonov, Sadye Paez, Rainer Melzer, Natalia Borowska-Zuchowska, Nicole Valenzuela, Daniela H. Palmer Droguett, Rita Monteiro, Petr Nguyen, Kamil S. Jaron, Daniel L. Jeffries, Caroline Howard, Tanja Schwander, Kerstin Howe, Ravinder K. Kanda, Arthur Georges, Wen-Juan Ma, Ore Francis, Mark Blaxter, Sarah P. Otto, Roman Hobza, Louise D. Heitzmann, Vasiliki Kousteni, Aurora Ruiz-Herrera, David Peris, James Umen, Cristiana Ramalho Maciel, Alexandr Sember, Ricardo Utsunomia, Yukako Katsura, Matthias Stöck, Simon Kershenbaum, Tymoteusz Pieszko, Vilma Loreto, Martha Mercy Mulongo. All contributors listed above gave their consent to be cited in the collaborative group of the Tree of Sex Consortium.

## Introduction

Reproduction is a defining characteristic of life. Whether sexual, clonal, or one of a myriad of strategies in between, every extant species has some successful method for propagation. Importantly, an organism’s **reproductive strategy** (see the glossary for all terms in bold) can influence all biological levels of organization, from genomes, cellular processes, development, physiology, through populations, ecology, behaviour, genetic diversity and the propensity for adaptation, diversification, and extinction. Describing the diversity of reproductive strategies among organisms and investigating how and why they evolve is thus essential when trying to understand the natural world.

Given the numerous biological factors associated with reproductive strategies, their characterization in diverse taxa requires multiple approaches and expertise from a broad range of disciplines, including genetics/genomics, cell biology, physiology, developmental biology, ecology, behavioural ecology, and evolutionary biology. Understanding the drivers and consequences of these strategies throughout nature requires subsequent in-depth analyses against a reliable phylogeny. However, before this is possible, information from disparate methodological and taxonomic disciplines must be integrated.

Roughly a decade ago, a group of scientists created the Tree of Sex database (Tree of Sex Consortium, 2014), collected data for over 37,000 species, across many eukaryotic lineages, and used it to gain several foundational insights into the distribution and evolution of reproductive strategies. These included the previously unrecognized lability and patterning of sex determination mechanisms across eukaryotes (Bachtrog et al., 2014), and well-supported estimates of variation in the rates of transitions among different reproductive strategies (Blackmon & Demuth, 2014; Blackmon et al., 2017; Pennell et al., 2018). Since then, extensive research and methodological advancements, including the continuing genomic revolution, have deepened our insights into reproductive biology. To capitalize on these advances, we have revived the Tree of Sex consortium. We are now working towards a new database that not only updates the information compiled by the first initiative, but also largely expands its scope to include all clades of eukaryotes for which data exists, all reproductive strategies identified to date, and new data types, including genomic data. Crucially, we will integrate this knowledge via a purpose-built ontology for reproductive biology, allowing us to connect information from disparate fields and from studies that are decades apart. The new Tree of Sex consortium will also serve as a framework for collaborative research to address fundamental questions about the evolution of reproductive strategies.

To set the stage for the new Tree of Sex project, here we summarize existing efforts to describe the diversity of eukaryotic reproductive strategies and highlight the current gaps in our knowledge and their importance. We then describe some of the topics and unanswered questions that we believe will benefit most from large comparative approaches that the Tree of Sex database will enable. Lastly, we describe our plans for the Tree of Sex database and the consortium, emphasizing the need for proactive engagement of a diversity of people, understandings, and perspectives so that we can build a database that encompasses the complexity and nuances of reproduction and its contexts throughout nature.

## Characterizing the diversity of reproductive strategies across eukaryotes

### Existing data on eukaryotic reproduction

Taxonomically speaking, the first Tree of Sex database (Tree of Sex Consortium, 2014) remains the broadest integrated collection of information on reproduction in eukaryotes compiled to date. In the decade since, several additional databases (Table 1) have been assembled, focusing on reproduction-related traits for distinct groups of organisms, including (but not limited to) fishes (Pla et al., 2022; Sember et al., 2021), amphibians and reptiles (Nemesházi & Bókony, 2023), insects (Chen et al., 2021), and plants (Baránková et al., 2020; Garcia et al., 2023). In addition, several other databases contain information that is relevant but not necessarily specific to reproduction, including karyotypes (Blackmon & Demuth, 2015a; Blaxter et al., 2024a; D’Ambrosio et al., 2017; Garcia et al., 2012; Jankásek et al., 2021; Román-Palacios et al., 2021; Sochorová et al., 2018) and genome sizes (Gregory, 2024; Pellicer & Leitch, 2020). Because reproductive strategies depend on and shape genomic characteristics (e.g., divergence, sex chromosomes, karyotype), these data types can greatly improve our understanding of the reproductive strategy of a given species.

**Table 1.** Example of existing databases containing data relevant to reproduction. The species numbers were derived from publications or directly by counting unique species names within available databases. Except for Tree of Sex v1, the databases are listed in alphabetical order.

Database name	Scope	Taxonomic scope	Species number	Reference
Tree Of Sex v1	Sexual systems, sex chromosomes	Vertebrates, invertebrates, plants	37,496	(Tree of Sex Consortium, 2014)
Animal Chromosome Count, ACC	Chromosome counts	Animals	14,524	(Román-Palacios et al., 2021)
AmphibianKaryo	Sex chromosomes, chromosome counts	Amphibians	2,124	(Perkins et al., 2019)
AndrodioecyAnimal	Androdioecy	Animal	36	(Weeks et al., 2006)
Animal_rDNA	Chromosome counts, karyotypes, genome size	Animals	2,800	(Sochorová et al., 2018)
AnimalGenomeSize	Genome size (C-values)	Animals	6,534	(Gregory, 2024)
ASER	Sex reversal	Animals	18	(Y. Li et al., 2021)
B-chrom	B chromosomes	Animals, plants, fungi	2,951	(D’Ambrosio et al., 2017; Gutiérrez et al., 2023)
CCDB	Chromosome counts	Plants	77,958	(Rice et al., 2015)
ChromNematoda	Chromosome counts, reproductive strategy	Nematodes	257	(Blaxter et al., 2024a)
ColeopteraKaryo	Sex chromosomes, chromosome counts	Beetles	4,960	(Blackmon & Demuth, 2015a)
DipteraKaryo	Sex chromosomes, chromosome counts	Diptera	3,443	(Morelli et al., 2022)
DrosophilaKaryo	Sex chromosomes, chromosome counts	Drosophila	1,247	(Morelli et al., 2022)
FishBase	Reproduction mode, life-history traits	Fish	35,400	(Froese & Pauly, 2024)
FISHKARYOME	Sex chromosomes, chromosome counts	Fish	1,285	(Nagpure et al., 2016)
FishSexChrom	Sex chromosomes (direct comparison with ToS v.1)	Fish	440	(Sember et al., 2021)
HarvestmenCyto	Chromosome counts	Harvestmen	100	(Tsurusaki et al., 2022)
HerpSexDet	Sex determination, sex reversal	Amphibians, reptiles	891	(Nemesházi & Bókony, 2023)
HZmatingSystem	Mating systems	Plants (hybrid zones)	245	(Pickup et al., 2019, 2020)
Insect_Egg_Evolution	Egg size and shape	Insects	6,700	(Church et al., 2019)
InSexBase	Sex chromosomes, sex-biased genes	Insects	49	(Chen et al., 2021)
KaryoBlatto	Chromosome counts	Termites, cockroaches	229	(Jankásek et al., 2021)
Mammal_SameSexSexualBehavior	Observed same-sex sexual behavior	Mammals	322	(Gómez et al., 2023)
MammalKaryo	Sex chromosomes, chromosome counts	Mammals	1,440	(Blackmon et al., 2019)
NESCent	Mating systems	Plants, life-history traits	154	(Goodwillie et al., 2005b, 2010)
Phaeodev	Genome sizes, ploidy	Brown algae	67	(The Phaeoexplorer project, 2024)
Phycocosm	Genome sizes	Algae, heterokonts	154	(Grigoriev et al., 2021)
Plant_DNA_C-values	Genome sizes (c-values), chromosome counts	Plants	12,273	(Leitch et al., 2019; Pellicer & Leitch, 2020)
Plant_rDNA	Chromosome counts, karyotypes, genome size	Plants	2,770	(Rodríguez-González et al., 2023)
PolyneopteraKaryo	Sex chromosomes, chromosome counts	Polyneoptera	823	(Sylvester et al., 2020)
PseudoscorpionCyto	Chromosome counts	Pseudoscorpions	65	(Šťáhlavský, 2022)
ReprodTraitsAlgae	Sexual systems, life-history traits	Brown algae	91	(Heesch et al., 2021)
ROSIE	Sex determination	Testudines	125	(Krueger & Janzen, 2022)
SAGD	Sex-biased genes	Animals	21	(Shi et al., 2019)
ScorpionCyto	Chromosome counts	Scorpions	264	(Schneider et al., 2024)
Sex-chrom	Sex chromosomes, ploidy, chromosome counts	Land plants, green algae	229	(Baránková et al., 2020; Garcia et al., 2023)
SexChromAlgae	Sex chromosomes	Brown algae	10	(Barrera-Redondo et al., 2024)
SexSystemCrustacea	Sexual systems	Crustaceans	334	(Benvenuto & Weeks, 2020)
SexSystemFish	Sexual systems	Fish	4,614	(Pla et al., 2022)
SpermTree	Sexual systems, sperm morphology	Animals	5,675	(Fitzpatrick et al., 2022)
SpiderCyto	Chromosome counts	Spiders	933	(Araujo et al., 2024)

Together, the above databases contain information for tens of thousands of species, including the presence or absence of sexual reproduction, mating systems, sex allocation, sex determination, karyotype, sex chromosome differentiation, ecological and behavioural phenotypes and many other traits that can inform the study of reproduction. However, these datasets are currently difficult to use in combination, as they lack standardized terms. Moreover, the lack of a common phylogeny hampers evolutionary analyses outside of the taxonomic bounds of each individual database.

### Gaps in our knowledge of reproduction in eukaryotes

**Figure 1.**
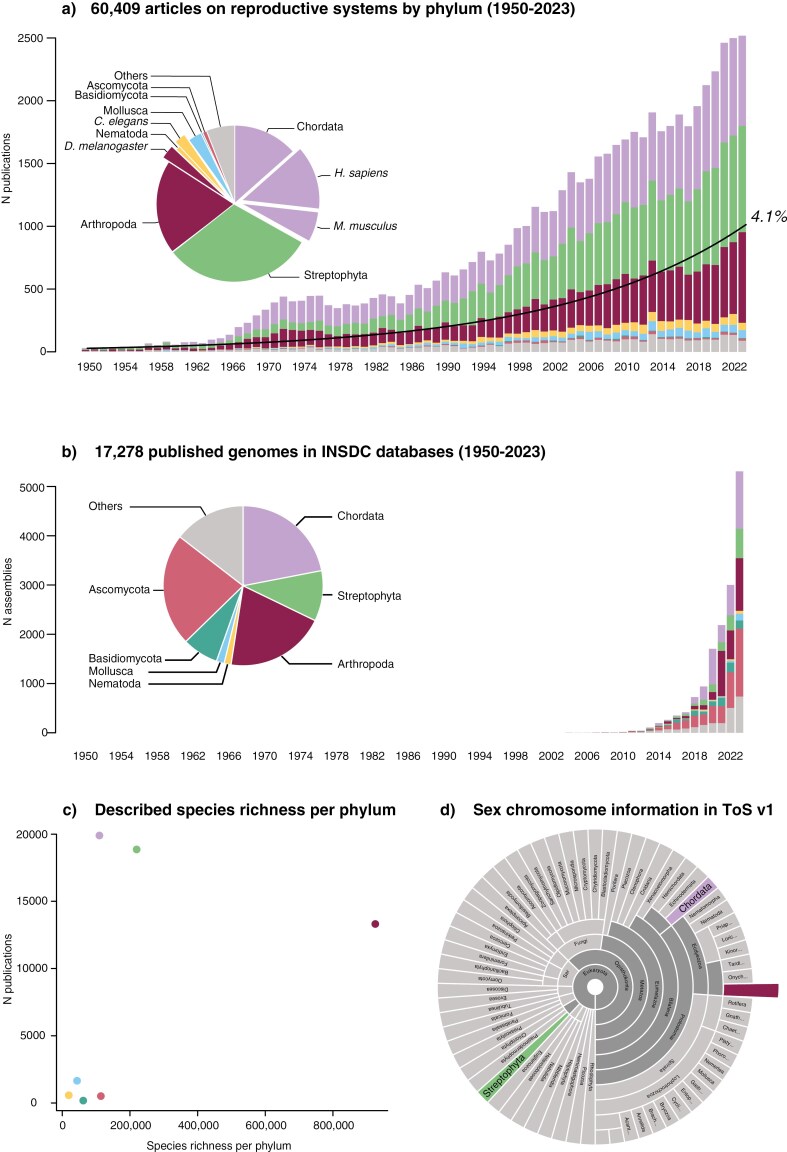
Summary of reproductive strategies literature and high-quality genome assemblies across eukaryotes. (A) Published primary literature by phylum and year. Literature libraries were searched using Dimensions (on 8th May 2024), with a search prompt that included 36 terms relating to topics within reproductive strategies research (search prompt: hermaphrodite OR monoecy OR dioecy OR gynodioecy OR androdioecy OR gynomonoecy OR andromonoecy OR polygamodioecy OR polygamomonoecy OR apomictic OR gonochorous OR parthenogenetic OR sex chromosome OR sex determination OR arrhenotoky OR haplodiploid OR genome elimination OR hybridogenesis OR dosage compensation OR self compatibility OR self incompatibility OR hermaphrodism OR monoecious OR dioecious OR gynodioecious OR androdioecious OR gynomonoecious OR andromonoecious OR polygamodioecious OR polygamomonoecious OR apomixis OR gonochoristic OR hermaphroditic OR parthenogenesis OR self compatible OR self incompatible). The resulting 85,533 papers were searched for taxonomic names taken from the NCBI taxonomy (downloaded 8th May 2024). A total of 60,409 could be classified into phyla (though no manual curation was performed, so some errors may exist). We included all name categories (e.g., scientific, common, blast names) for all taxonomic ranks between phylum and genus. Common names and blast names were also included for species, but species scientific names were not used to reduce the total number of search terms, and because genera were already included. Inset pie chart shows the total proportion of all classified papers by phylum, with four model organisms also shown. For comparison, the black line shows a 4.1% annual increase publication rate estimated as the rate for all scientific publications (Bornmann et al., 2021) (B) All genomes archived by the The International Nucleotide Sequence Database Collaboration by phylum and year (Created in GoaT (Challis et al., 2023) on 06 June 2024). Only one assembly per species is counted. (C) Estimate of the number of named species for the 15 most speciose phyla taken from the Global Biodiversity Information Facility as of 16th May 2024. (D) Distribution of sex chromosome information (morphology and system of heterogamety) from ToS v1 database across eukaryotic phyla (Created in GoaT from data sources here).

Despite extensive efforts, existing databases capture only a portion of the existing literature, which itself does not encompass the full diversity of reproductive strategies in nature. To identify taxonomic gaps in our knowledge, we conducted a literature search based on a non-exhaustive set of 36 keywords related to reproductive strategies (see legend of Figure 1). The search hits reveal a substantial taxonomic bias in the literature on reproductive strategies (Figure 1A). Unsurprisingly, species with relevance to medicine, agriculture, and basic research (such as humans, mice, fruit flies, and the roundworm *Caenorhabditis elegans*) have received the most research interest. Indeed, over half of all papers studying chordates focus on humans or mice. Consequently, the spread of research across individual phyla does not reflect their species richness (Figure 1C).

One additional factor likely contributing to these taxonomic biases is the difficulty of characterizing reproductive strategies in non-model organisms. However, technological advancements are mitigating many such issues. Genomic techniques can help characterize the ploidy, karyotype, inheritance, sex chromosomes, sex-specific gene expression patterns, population dynamics, and other reproduction-relevant traits, in many cases without any prior knowledge. While it is still necessary to integrate such data with ecological, phenotypic, quantitative genetics, and cytogenetic approaches in order to gain a holistic picture of reproduction in a given species, genomics can provide a valuable foundation on which to build our understanding of reproduction, even in hard to study organisms (Deakin et al., 2019; Stöck et al., 2021a; Zaidem et al., 2019).

In addition to biases from methodological limitations or applied practices, it is well known that the attention received by a given study system in biology is also affected by cultural and geographical biases. Owing to differences in resources and funding access, the most biodiverse regions of the planet have received the least research focus (Linck & Cadena, 2024; Titley et al., 2017). This also affects the diversity of researchers studying a given species as well as phrasing of hypotheses (Ahnesjö et al., 2020). Despite the ever-increasing yearly publication rates across science (Bornmann et al., 2021), the taxonomic biases in the distribution of publications on reproductive strategies per phylum have remained stable for over 70 years (Figure 1A). The available genomes show a different skew (Figure 1B) to that of the published literature, however, this has again changed little through time. Together, the stability of these trends highlights that closing the taxonomic gaps in our knowledge will require proactivity in the choice of future study systems and a serious push for inclusive research strategies that provide opportunities to researchers in low- and middle-income countries.

When defining a strategy to mitigate problems caused by existing biases in study systems, one important factor to consider is the reproductive diversity of the studied taxa. For example, the large majority of studied viviparous mammals possess an XX/XY sex determination system with Sry as a sex determining gene, which has remained conserved for hundreds of millions of years (Hughes & Page, 2015). As such, it is less likely that exploring additional viviparous mammal systems will significantly broaden our knowledge of diversity and frequency of sex determination systems in eukaryotes. In contrast, based on the species studied so far, animals such as amphibians (Jeffries et al., 2018; Ma & Veltsos, 2021), reptiles (Gamble et al., 2015; Holleley et al., 2015; Krueger & Janzen, 2022; Pinto et al., 2022, 2023), fish (El Taher et al., 2021; Jeffries et al., 2018; Kitano et al., 2024), crustaceans (J. Li, 2022; Ye et al., 2023), insects (Blackmon et al., 2017; Bracewell et al., 2024; Vicoso & Bachtrog, 2015), arachnids (Araujo et al., 2012; Cordellier et al., 2020; Kořínková & Král, 2013), and potentially molluscs (Breton et al., 2018; Yusa, 2007) seem to display a large diversity of sex determination mechanisms, and yet they have received comparatively little attention in this regard. Similarly, haploid sexual systems are severely understudied, despite being found in many taxa including algae and bryophytes (Charlesworth, 2022; Coelho et al., 2018) and being important for testing predictions about sex chromosome evolution. Finally, there are many more fundamental gaps in our knowledge of reproduction of unicellular eukaryotes. For example, in microsporidia (important spore-forming unicellular fungi that parasitize all major groups of animals) all we know is that some form of sexual reproduction likely occurs, based on the presence of an intact multi-gene cassette involved in mating-type determination in fungi (Lee et al., 2008), and putatively imaged gametes (reviewed in Khalaf et al., 2024). These taxa, which have traditionally been overlooked and show signs of high variability in reproductive strategies, likely hold the most potential for broadening our knowledge of reproductive strategy diversity and understanding the relative frequencies of the many systems that exist in nature.

We note, however, that we are not petitioning against the further study of already-well-studied clades. Indeed, the ideal study system is defined by the question at hand, and the in-depth study of model systems, and those that are exceptions to clade norms can be extremely insightful for specific evolutionary processes, as has been the case with the few exceptional mammal species which do not share the usual mammalian XX/XY sex determination system (e.g., Fredga, 1988; Hughes et al. 2024 ; Mulugeta et al., 2016; Ruiz-Herrera & Waters, 2022; Saunders & Veyrunes, 2021).

Aside from taxonomic gaps, the types of data available for a given study system are also sporadic, with many lacking cytological (e.g., chromosome morphologies, centromere positions) or genomic data. The combination of high-throughput genomics with cytogenetic analyses (as in Deakin et al., 2019; Liehr, 2021) will undoubtedly prove to be a powerful approach to studying patterns of chromosomal rearrangements, such as inversions, translocations, fusions, or fissions, which can all impact reproductive processes including recombination in meiosis or gametogenesis, e.g., resulting in complete versus incomplete genome complements in gametes (Baránková et al., 2020; Deakin et al., 2019; Gil-Fernández et al., 2020; Vara et al., 2021), as well as speciation and hybridization. Such approaches will also be instrumental in describing phenomena such as programmed DNA elimination, which leads to differences in (sex) chromosome numbers or synteny between germline and somatic genomes. While **programmed DNA elimination** has so far been observed in multiple eukaryotic lineages (Hodson & Ross, 2021; Smith et al., 2021; Wang & Davis, 2014), its frequency is still not well known.

Advancements in chromosome conformation capture (“3C”) methods, such as Hi-C (Jerkovic & Cavalli, 2021; Rao et al., 2014), are also opening new avenues for studying genome evolution across various timescales and cell types (Álvarez-González et al., 2022; Bista et al., 2024; Hoencamp et al., 2021; Vara et al., 2019). This technology has already provided insights into the chromatin structure of sex chromosomes in both somatic and germ cells, albeit in a limited number of organisms (Bista et al., 2024; Chen et al., 2020; Hoencamp et al., 2021; Liu et al., 2024; Vara et al., 2019). As the accessibility of these approaches increases, so will our understanding of the role of chromatin architecture during meiosis and thus its role in the evolution of reproductive mechanisms (e.g., Álvarez-González & Ruiz-Herrera, 2025; Chen et al., 2020).

In summary, methodological advances like those listed above promise exciting opportunities for closing existing gaps in our knowledge of reproduction. The Tree of Sex consortium is thus committed to accommodating any such data type that could be pertinent to the interpretation of reproductive diversity to facilitate a holistic understanding of reproduction.

## Comparative analyses and their role in advancing the field of reproductive strategy evolution

Centralizing and integrating information across the field will enable large, phylogenetically deep comparative analyses to test for the evolutionary forces that shape diversity in reproductive strategies. With such analyses we can estimate the rate of reproductive trait evolution, the likelihood of transitions from one strategy to another, test whether trait evolution or transitions correlate with environmental, ecological, physiological, or genetic changes, and reconstruct the reproductive strategies in the ancestors of extant taxa. By compiling information as exhaustively as possible across eukaryotes, the Tree of Sex database will facilitate analyses at a previously unfeasible scale and resolution. Below we highlight several broad topics and ways in which we envisage such comparative analyses might advance our knowledge of reproduction.

### The evolution of sexual vs asexual reproduction, and everything in between

**Figure 2.**
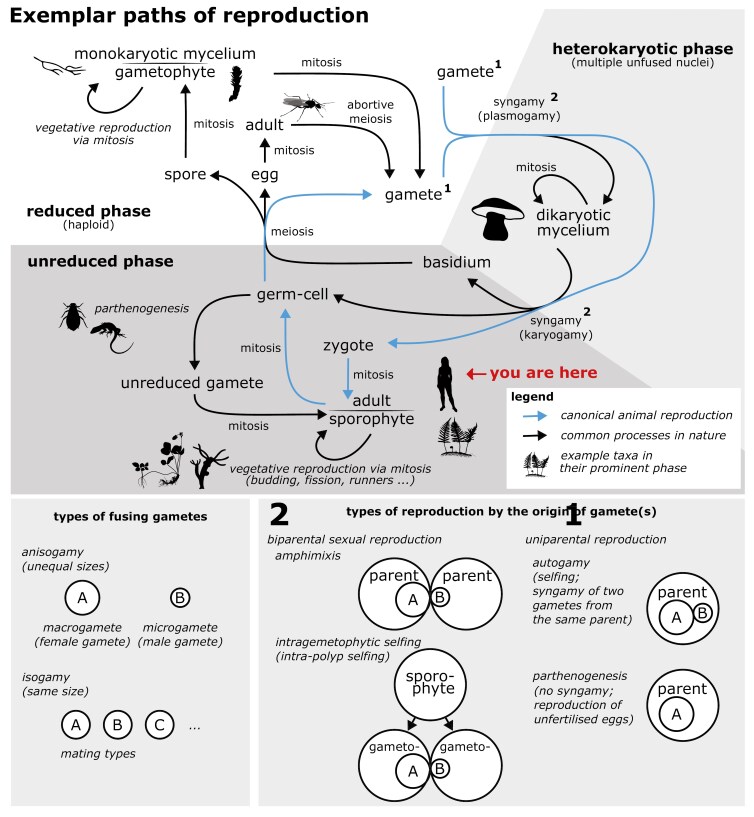
Selected examples of diverse reproductive strategies found in eukaryotes. The canonical animal reproductive cycle is highlighted in blue. The prominent phase of life is indicated by the following taxa/silhouettes listed here clockwise starting in top-right corner: Ascomycota (generic hyphae), Bryophytes (sporophyte of *Rosulabryum capillare*), Hymenopterans (male *Camponotus* sp.), Basidiomycota (*Boletus edulis*), animals (*Homo sapiens*), vascular plants (*Polypodium vulgare*), cnidarians (budding *Hydra vulgaris*), vascular plants (runner of *Fragaria* sp.), aphids (generic member of Aphididae), and Caucasian rock lizards (*Darevskia* spp.). The majority of animals or vascular plants spend most of their lives in the unreduced (diploid or polyploid) state, while the dominant phase in bryophytes is haploid, and algae span a diversity of life cycles. Plants and animals fuse gametes (plasmogamy) and nuclei (karyogamy) very quickly within a process we jointly call syngamy, though this is not universally true. For instance, basidiomycete fungi live for a substantial part of their lives as large fruiting bodies of dikaryotic cells (with two unfused nuclei). In other cases, such in haplodiploid species, males are haploid, develop from unfertilized eggs and generate sperm via abortive meiosis. Some species can also reproduce via unfertilized gametes (parthenogenesis) or by vegetative (asexual) reproduction during their haploid and/or diploid phases. Parthenogenesis frequently features a modified meiosis, or a regular meiosis with pre- (endoreplication) or post- (gamete duplication) meiotic genome duplication restoring the parental ploidy. This figure includes only a small selection of the many paths that reproduction can take, which were chosen to highlight some of the major differences in reproductive strategies in eukaryotes.

**Sexual reproduction**, broadly viewed as a process that brings together and mixes the genetic material from two parental gametes, is widespread in eukaryotes (Speijer et al. 2015) . At its core, sexual reproduction involves the production of haploid (reduced) cells via meiosis. These cells can either be gametes (in most animals), or spores that will develop and produce gametes after additional mitotic cell divisions (in land plants). Haploid gametes then fuse (**syngamy**) during fertilization to create the genome of the offspring. However, within this broad definition of sexual reproduction lies a large diversity of strategies (Figure 2). For example, sexual reproductive strategies can differ in relative time spent within individual stages of their cycle. In some cases, the reduced stage is dominant; in others, the unreduced stage is dominant, while others spend large portions of their life cycle between these stages in the heterokaryotic phase. Strategies also differ in the types of gametes or spores that they produce. Many sexual eukaryotes are **anisogamous**, producing two gamete types that differ in size and form (e.g., eggs and sperm in many animals), while others are **isogamous**, wherein all gametes produced are of similar size and form (e.g., brewer’s yeast and some protists, dinoflagellates, and algae (Lehtonen et al., 2016)). A similar distinction can be made between spore-producing organisms which can either make spores of similar size (i.e., isospory, found in bryophytes; Mogensen 1985 and most ferns; Dyer 1979 ) or different sizes (anisospory, as found in seed plants; Jesson & Garnock-Jones 2011). Lastly, in some species, syngamy occurs between the gametes from two individuals (biparental sexual reproduction); in others, zygotes can be made from the fusion of gametes from only one individual (uniparental sexual reproduction), and some species can do both.

The evolution of anisogamy has received much attention in the context of species with distinct gamete types carried by different individuals (Parker, 1978). Anisogamy is thought to be a major cause of the evolution of different sex phenotypes (Schärer et al. 2012), and of sexual selection (Janicke et al., 2016). But why does anisogamy evolve despite its tendency to restrict mating opportunities (Otto, 2009)? And why is the predominant number of gamete classes two (Charlesworth, 1978)? Many models have been proposed to answer these questions (Billiard et al., 2011), with some receiving theoretical support (Constable & Kokko, 2021; Lehtonen & Kokko, 2011; Lehtonen & Parker, 2014; Lehtonen et al., 2021). However, conclusive empirical evidence for any of them remains rare. One type of empirical evidence that could prove useful in this case is the association of gamete types with other fundamental reproductive traits (e.g., self-compatibility, sex ratios, gamete competition/fertilization success) though such analyses would require multiple phylogenetically independent taxa to provide statistical power. Thus, this topic could benefit greatly from the integrated knowledge of such traits across eukaryotes, and especially clades such as algae (Coelho et al., 2018; Krueger-Hadfield, 2024) and fungi (Billiard et al., 2011).

The two distinct types of gametes of anisogamous species can be produced by different individuals or within a single individual. When produced by different individuals, we refer to the species as **gonochoristic** in animals, or **dioecious** in plants with a diploid-dominant life cycle. While in plants with a haploid-dominant life cycle, species in which different gamete types are produced by separate gametophytes are termed **dioicous** (Villarreal and Renner 2013) (Figure 2A). If both gamete types are produced by a single individual, we refer to them as **hermaphroditic/monoecious/monoicous**. Hermaphrodites can further be classified into those in which individuals can produce both types of gametes at the same time (**simultaneous/synchronous hermaphroditism**) or at different times (**sequential hermaphroditism**). In plants, **hermaphroditism** refers to systems where individuals carry bi-sexual flowers, while the system where individuals generate male and female flowers is called **monoecious.** Hermaphroditism is the most common sexual system in flowering plants (Käfer et al., 2017; Renner 2014) and has been described in a variety of invertebrates (see initial review by Ghiselin, 1969); while among vertebrates, it is thought to be specific to teleost fishes (Pla et al., 2022). Hermaphrodites can also coexist with males (androdioecy), females (gynodioecy) or both (trioecy/trioicy), in mixed sexual systems. In animals (Sasson & Ryan, 2017; Weeks et al., 2006), androdioecy is known in very few taxa, for example, in *Caenorhabditis* nematodes (Thomas et al., 2012), in branchiopod crustaceans (Benvenuto & Weeks, 2020); in barnacles (Ewers-Saucedo et al., 2016) and among vertebrates, it has only been observed in *Kryptolebias* killifishes (Costa et al., 2010). Gynodioecy is even more rare in animals (Weeks, 2012). In flowering plants, gynodioecy is more common than androdioecy (McCauley & Bailey, 2009), while trioecy is very rare in both animals (Oyarzún et al., 2020) and flowering plants (Godin, 2022). Recent studies have investigated evolutionary transitions among all sexual systems (Goldberg et al., 2017; Leonard, 2019; Pannell & Jordan, 2022; Weeks, 2012). By centralizing all known examples of sexual systems and self-compatibility in eukaryotes, the Tree of Sex database will allow us to better test whether the contexts of these strategies are general across Eukaryota.

While sexual reproduction is often thought of as involving two individuals (biparental), in **self-compatible simultaneous hermaphrodites** the fusing gametes might be from the same parent in a process known as self-fertilization or selfing. Selfing is found in plants (Baker, 1955; Barrett, 1998; Hedrick, 1987) and animals (Goodwillie et al., 2005a; Jarne & Auld, 2006; Jarne & Charlesworth, 1993), in **automictic** fungi (Hood & Antonovics, 2004) and algae (Chepurnov et al., 2004; Heesch et al. 2021; Krueger-Hadfield et al., 2024). Uniparental sexual reproduction has been most extensively studied in the form of self-fertilization in plants (Cheptou, 2018; Hartfield et al., 2017; Wright et al., 2013), which is often thought to evolve in response to selection for reproductive assurance when mates (or pollinators) are scarce (Baker, 1955; Pannell et al., 2015). Selfing in animals has been reported in a variety of invertebrates (Annelida, Arthropoda, Cnidaria, Echinodermata, Ectoprocta, Mollusca, Nematoda, Platyhelminthes, and Urochordata; Jarne & Auld, 2006). Isogamous species might undergo self-fertilization via fusion of gamete of the same meiotic tetrad (Billiard et al., 2011). Unfortunately, many selfing taxa, e.g., algae, still lack population genetic data with which to test theories for the predictors and consequences of selfing (Krueger-Hadfield, 2024). However, by centralizing known examples of gonochorism, hermaphroditism, and self-fertilization across diverse lineages, the Tree of Sex database will allow us to search for explanations for the distribution of selfing across eukaryotes.

Uniparental reproduction might also happen without fusion of gametes (syngamy), which is known as **parthenogenesis**. These species usually have a modified meiosis or pre- or post-meiotic genome duplication, resulting in unreduced gametes and thus to stable ploidy levels between generations (Stöck et al., 2021b). The offspring might be a genetic clone of the parent (e.g., in parthenogenetic animals using premeiotic endoreplication; Dedukh et al., 2024) or a mixture of parental haplotypes known in plant and animal literature as **automixis**. Finally, reproduction can occur via separation of somatic tissue such as vegetative propagation in plants (Schwaegerle, 2005), budding or fragmentation in cnidarians (Berzins et al., 2021), fissiparity in some flatworms (Zaccanti & Farne, 1986) and echinoderms (Dolmatov et al., 2018; Thuy et al., 2024).

Though we have presented sexual and asexual reproduction above as separate modes of reproduction, in fact a spectrum of strategies exists, which do not neatly fall into these two discrete categories. For example, in sperm-dependent parthenogenesis, also known as gynogenesis, sperm is required but only to initiate embryo development, i.e., its DNA is not incorporated into the zygote (Beukeboom & Vrijenhoek, 1998). This system is known, for example, in the teleost fish genera *Poecilia* and *Poeciliopsis* (Cerepaka & Schlupp, 2023; Lampert & Schartl, 2008; Schlupp, 2005; Schultz, 1969)*, Carassius* (Choleva et al., 2008; Komen & Thorgaard, 2007; Liet al., 2018) and *Cobitis* (Choleva & Janko, 2013). Similarly, androgenesis requires two parents for fertilization, but a zygote is formed solely with paternal nuclear genes; this is found in plants and animals (Schwander & Oldroyd, 2016). Some amphibians, teleosts and insects perform hybridogenesis, wherein DNA from both sperm and egg are incorporated into the zygote; however, the genome of one of the parental species is consistently eliminated during meiosis in each generation (Neaves & Baumann, 2011).

The categorization of organisms as exclusively sexual or asexual also does not work for organisms using more than one reproductive strategy, as in cyclical parthenogenesis (found in aphids and water fleas) or **arrhenotoky** (a form of haplodiploidy) where males develop parthenogenetically from unfertilized eggs and females develop sexually from fertilized egg (for example in hymenopterans, thrips, and several other arthropod lineages; reviewed in de la Filia et al., 2015). Finally, many species switch between sexual and asexual forms of reproduction. Such strategies are used by most studied unicellular eukaryotes which generally reproduce asexually, with occasional sexual cycles (Green & Noakes, 1995; Hofstatter & Lahr, 2019).

Obligately asexual (or uniparental) reproduction is relatively rare, which has led to the famous question “why sex?” (Bell, 1981; Hartfield & Keightley, 2012; Otto, 2009, 2021). Indeed, where, how, and why sexual reproduction and its constituent processes (meiotic recombination, outcrossing, anisogamy, and sex) have evolved or been lost has received much attention. It is thought that recombination dates back to the ancestor of all eukaryotes (Bernstein & Bernstein, 2010; Mirzaghaderi & Hörandl, 2016; Ramesh et al., 2005). Yet, recombination has been lost or much reduced in the meiosis of the heterogametic sex in many species (Morgan, 1914; Sardell & Kirkpatrick, 2020; Satomura et al., 2019), or lost entirely in some asexuals employing asynaptic meiosis, like the Amazon molly (Dedukh et al., 2022) or achiasmatic meiosis, as in *Bithynia* snails (Debus, 1978) and scorpions (Shanahan & Hayman, 1990). It has been proposed that recombination evolved because it disrupts detrimental combinations of alleles and brings together beneficial alleles that arise in different individuals (Felsenstein, 1974; Hartfield & Keightley, 2012; Otto, 2009). Furthermore, recent theoretical work points to selective interference as the main driver of the evolution of sexual reproduction with recombination (Otto, 2021). Without genetic mixing and recombination, selection cannot act upon individual loci, as genetic variance is restricted to the level of the whole genome. There is substantial empirical support for selective interference (Neiman et al., 2018); however, so far it is restricted to individual genes and limited taxonomically. Importantly, many of these hypotheses have remained largely untested, primarily due to technical challenges in accurately estimating key parameters, such recombination rates, effective population sizes, and selection coefficients (Dapper & Payseur, 2017), as well as the fact that sex and meiotic recombination evolved only a small number of times. Thus, there is yet to be a fully satisfactory explanation for the vast prevalence of sex, which necessarily needs to be based on a study of a taxonomically diverse set of asexual species.

The complexities of categorizing reproductive strategies make comparative analyses difficult. By structuring the database to include nuanced characteristics of meiosis and inheritance for these species, the Tree of Sex will allow us to study the evolution of various components of these reproductive strategies in their most relevant context without forcing them into categories that could mask important patterns.

### Explaining the many ways of sex determination

Anisogamy is more common than isogamy among multicellular eukaryotes and is often associated with dioecy/dioicy/gonochorism. In such species, mechanisms must exist to determine which type(s) of gamete an individual will produce, and individuals are classified as female (producing macrogametes) or male (producing microgametes). We refer to such mechanisms as sex determination (Beukeboom & Perrin, 2014; Bull, 1983). This term is also sometimes used in isogamous clades such as algae and fungi, though, to avoid confusion, we refer to individuals producing different types of similarly sized gametes as **mating types** (Aanen et al., 2016).

One of the main goals of the first Tree of Sex project was to synthesize the many ways in which sex is determined across eukaryotes and the non-random distribution of these mechanisms among eukaryotic clades (Bachtrog et al., 2014). For instance, to our knowledge every mammal or bird has **genotypic sex determination (GSD)**, wherein the sex of an individual is determined by their genotype at one or more locus, or in their karyotype. In contrast, some reptiles and teleost fishes use **environmental sex determination (ESD)**, wherein male or female development depends on environmental cues (e.g., temperature or social environment).

Similarly to reproductive strategies, however, sex determination modes resist binary classification into GSD or ESD with sex in some species being determined by the interaction between genotype and the environment (Baroiller et al., 1995; Collin, 2006; Holleley et al., 2015; Mork et al., 2014; Piferrer et al., 2005; Sarre et al., 2004; Whiteley et al., 2021). In addition, there are increasing examples of putative GSD systems in which the genotype at the presumed sex determination locus is less predictive of the sex phenotype than originally thought (Cossard & Pannell, 2019; Ehlers & Bataillon, 2007; Holleley et al., 2016; Nemesházi & Bókony, 2022; Nemesházi et al., 2020; Phillips et al., 2020; Rodrigues et al., 2018; Wiggins et al., 2020). It has been proposed (but not yet empirically tested) that such cases could arise from interactions between the random noise inherent in gene expression and the required expression thresholds for sex development pathways (Perrin, 2016). Though, it is also possible that the occurrence of mismatches between genotypic and phenotypic sex could be favoured by natural selection as it can allow for occasional self-fertilization, which could be beneficial in some situations (e.g., low mate abundance, Crossman and Charlesworth 2014; Pannell 2015).

There is also ample variation in sex determination mechanisms within GSD systems. In many cases a single locus acts, in a switch-like fashion, to divert development to the male or female pathway. This gene is widely referred to as the Master Sex Determination gene, though some suggest that it should be referred to as the Primary Signal (PS) gene reflecting a more polygenic view of developmental regulation (Kocher et al., 2024). We use the latter from hereon. Polygenic sex determination has indeed been shown in some species, such that multiple genes in an individual’s genome act together to determine sex (reviewed in Kocher et al., 2024; Schartl et al., 2023). Another form of a GSD present in several diverse taxa (15% of known arthropod species; de la Filia et al., 2015) is arrhenotoky, which has independently evolved multiple times (Normark, 2003; Ross et al., 2015) and is often associated with eusociality (da Silva, 2022; Joshi & Wiens, 2023), inbreeding (Hamilton, 1967; Ross et al., 2015), flexible sex allocation (Hamilton, 1967; Normark, 2004), reproductive manipulation by infectious endosymbionts (Werren et al., 2008) and often uses the ecologically vulnerable complementary sex determination system (Beye et al., 2003; Leung & van der Meulen, 2022; Zayed & Packer, 2005).

Despite great progress in the discovery of sex determination mechanisms across eukaryotes (Bachtrog et al., 2014; Beukeboom & Perrin, 2014; Blackmon et al., 2015, 2017; Goldberg et al., 2017; Iwasaki et al., 2021; Sabath et al., 2016), this information is distributed in a very biased way across eukaryotic clades. For example, we currently know the PS gene of approximately 140 teleost species (Kitano et al., 2024; Wang et al., 2024), which has allowed us to recognize not only the high amounts of polymorphism in PS genes in this clade, but also the strong convergence on a few genes, implying strong evolutionary constraints on which genes can become PS genes in some lineages. These insights highlight how important and powerful it is to have good phylogenetic coverage and detailed information on reproductive strategies. In contrast, we currently have no knowledge of the sex determination genes in most organisms, including even well-studied ones like guppies (Charlesworth et al., 2020) or *Rumex* plants (Sacchi et al., 2024) to name but two cases. This not only precludes similar inferences in these clades but also prevents our understanding of whether the evolutionary patterns seen within teleosts are an exception, or whether they are indicative of a general pattern across eukaryotes. This issue applies not only to the study of PS genes, but also to mechanisms of ESD, which, to our knowledge have only been described to date in turtles (Ge et al., 2018; Schroeder et al., 2016; Weber et al., 2020) and *Daphnia* (Kato et al., 2024), though similar ESD mechanisms involving thermally sensitive isoforms have been implicated across distantly related reptiles (Deveson et al., 2017; Whiteley et al., 2022).

By integrating available data on sex determination systems across eukaryotes, the Tree of Sex database will facilitate comparative approaches to describe, with higher resolution than before, the distribution of sex determination mechanisms across eukaryotes, their association with relevant traits (e.g., life cycle, environmental stability), and patterns of gene use for sex determination.

### Sex chromosome evolution

In organisms with GSD, the term sex chromosome refers to a linkage group harbouring a locus whose inheritance correlates with an organism’s sex. They are common among eukaryotes with separate sexes and have evolved many times independently across many clades (Bachtrog et al., 2011; reviewed in Bachtrog et al., 2014). The term “sex chromosome” refers to all ranges of sex chromosome differentiation (see below), including systems in which one homolog is almost completely sex linked, completely absent, or in which the sex-linked region containing the sex determination gene represents only a small portion of the chromosome, with the rest segregating autosomally (**pseudoautosomal region [PAR]**). In organisms with fixed separate sexes, in which sex is determined in the diploid phase of the life cycle, sex chromosome systems can be split into two major categories: (1) male **heterogamety** (e.g., XX/XY), where males (XY) can produce two different (hence “hetero”) types of haploid gametes, containing either an X or a Y chromosome; and (2) female **heterogamety** (e.g., ZZ/ZW), where it is the females that produce two different gamete types (e.g., with either a Z or a W chromosome). In such systems, the sex-limited chromosome may carry an allele that initiates development of the heterogametic sex, or sex might be determined by the number of X (Bridges, 1916; Meyer, 2022) or Z (Ioannidis et al., 2021) chromosomes. In some organisms, no sex-limited chromosome exists (e.g., XX/X0 in nematodes (Nigon, 1949), springtails (Núñez, 1962), and many insects (reviewed in Blackmon et al., 2017; White, 1945), or ZZ/Z0 in many moths (Sahara et al., 2012)). Again, in such cases, it is the number of X or Z chromosomes that determines sex. And finally, taxa in which *only* the sex-limited chromosome exists have also been found (00/Y0, or 00/W0) (Jonika et al., 2022), though the latter is seemingly rare. In anisogamous species in which sexes are determined in the haploid life phase, sex chromosomes are denoted as **U/V**, with only one of the two being present in a given haploid cell (Ahmed et al., 2014; Lipinska et al., 2024; Nieuwenhuis & James, 2016). Finally, while isogamous species do not have sex chromosomes, their “mating-type chromosomes” often behave similarly to U/V chromosomes (Coelho et al., 2018).

One of the most notable and consistent properties of sex chromosomes is the tendency for recombination to be reduced between the sex-limited chromosome (e.g., Y or W) and its **gametolog** (e.g., X or Z) (Charlesworth, 2023; reviewed in Charlesworth et al., 2005). This can either be a progressive reduction (as reviewed in Jay et al., 2024), or perhaps due to a sex determination gene arising in a region that already lacked recombination (Bergero et al., 2019; Rifkin et al., 2020, 2022). Due to the lack of genetic exchange between gametologs, the sex-linked region often degenerates over time, losing much of its gene function and content (Muller, 1964) and accumulating repetitive elements (Charlesworth et al., 1994; Nguyen & Bachtrog, 2021; Peona et al., 2021). However, due to differences in selection in the two sexes, sex chromosomes can also become specialized, e.g., Y chromosomes may become enriched in genes important for male reproduction (Bachtrog et al., 2019; Carvalho et al., 2001; Colaco & Modi, 2018; Okada et al., 2001). In contrast to XY/ZW systems, the degeneration and/or specialization of U and V chromosomes is expected to be symmetrical as they are each inherited without recombination by gametophytes of just one sex (Bull, 1978; Carey et al., 2021; Immler & Otto, 2015).

The evolutionary forces driving recombination reduction on sex chromosomes have received considerable debate. Several theoretical models have been proposed to explain this process (reviewed in Charlesworth, 2023; Jay et al., 2024; Käfer, 2022; Ponnikas et al., 2018; Veltsos et al., 2024). Most of these theories are adaptive (but see Jeffries et al., 2021; Kent et al., 2017; Rifkin et al., 2020) and invoke selection either due to the presence of sexually antagonistic polymorphisms, deleterious mutations, regulatory evolution, or meiotic drive. While debate is ongoing as to which of these models is most promising (Bergero & Charlesworth, 2019; Charlesworth & Olito, 2024; Jay et al., 2024; Lenormand & Roze, 2024), this discussion is almost entirely theoretical and rests on many untested assumptions (summarized in Jay et al., 2024). However, some of these assumptions can potentially be tested (Charlesworth, 2023) and the Tree of Sex database will facilitate this, for example, by relating the rate and extent of recombination loss on sex chromosomes to its proposed drivers across phylogenetically distant species.

Another interesting characteristic of sex chromosomes is the rate at which fusions between sex chromosomes and autosomes fix within some lineages (Anderson et al., 2020; Lisachov et al., 2021; Pennell et al., 2015; Pokorná et al., 2014; Wright et al., 2024). This is often followed again by the spread of recombination reduction to parts of the fused chromosome (**neo-sex chromosome**), further increasing the proportion of the genome which is sex linked. In extreme cases, this can happen multiple times, as in the sex chromosome chains of monotremes (Rens et al., 2007). Proposed evolutionary drivers of such fusions overlap with those proposed above for recombination suppression. For example, a sex chromosome-autosome fusion may be favoured if the autosome harbours a gene under sexually antagonistic selection (Charlesworth & Charlesworth, 1980; Matsumoto & Kitano, 2016). However, additional hypotheses specific to the fixation of fusions also exist. The fragile Y hypothesis (Blackmon & Demuth, 2015b), for example, proposes that, as recombination is lost on a sex chromosome, opportunity for chiasmata is reduced along much of its length. As chiasmata are important for correct chromosomal segregation during meiosis, their absence can result in maladaptive or lethal aneuploidies. This could provide an advantage to sex chromosomes fused to an autosome, which could restore pairing. Here, the Tree of Sex database will facilitate a more complete characterization of the rates of various types of fusions across the eukaryotic phylogeny. Further, it will allow for tests of association between the rate of fusions and the extent of sex chromosome degeneration, size of the pseudoautosomal region, the integrity of centromeres, holocentricity, and many more potential predictors across a much more diverse set of lineages than is currently possible.

Extensive degeneration via deletions, massive accumulation of repeat elements (Chalopin et al., 2015), or fusions can all result in **heteromorphic** sex chromosomes, i.e., those that show morphological differences that can be seen via microscopy. As such techniques were the only ones available for decades after sex chromosomes were first discovered in 1905 (Brush, 1978; Stevens, 1905, 1906), heteromorphic sex chromosomes were generally the only ones that could be detected and studied. However, the increasing availability of genetic and genomic techniques has enabled the discovery of many new sex chromosome systems that do not show morphological differences via microscopy (**homomorphic**). While this newly available data is still fragmented, it has revealed that homomorphic sex chromosomes are common among eukaryotes (Bachtrog et al., 2014). Further, the distribution of homomorphic vs heteromorphic sex chromosomes across eukaryotic lineages is highly heterogeneous. For example, in mammals almost every species has heteromorphic sex chromosomes (Graves, 1995), while in amphibians, between 75% and 96% of species are thought to have homomorphic sex chromosomes (Ma & Veltsos, 2021; Schmid et al., 1991). Assembling detailed information for sex chromosome differentiation from all data types, including microscopy, genetic maps and genomic data within the Tree of Sex database will help crystallize this picture, and give a much more accurate view of the extent and distribution of sex chromosome degeneration across the eukaryote phylogeny.

The extent of sex chromosome degeneration observed is related to the frequency at which new sex chromosomes arise in each clade. Transitions from hermaphroditism to gonochorism (with GSD; Ming et al., 2011), ESD to GSD (Johnson Pokorná & Kratochvíl, 2016; Sabath et al., 2016), or from one GSD system to another (i.e., sex chromosome turnovers; El Taher et al., 2021; Gamble et al., 2015; Jeffries et al., 2018; Kratochvíl et al., 2021b; Vicoso, 2019) will all create new sex chromosomes. Because, in most cases, new sex chromosomes arise from existing autosomes (but see Fraïsse et al., 2017; Imarazene et al., 2021), and because sex chromosomes degenerate progressively through time, the gametologs of very young sex chromosome systems are generally undifferentiated. Lineages with frequent transitions in sex determination system thus tend to have undifferentiated sex chromosomes (Blaser et al., 2014; El Taher et al., 2021; Jeffries et al., 2018). However, correlations between the age of a sex chromosome system and levels of degeneration can be weak across divergent taxa (Renner & Müller, 2021) and there are several known cases of old yet homomorphic sex chromosome systems (Kuhl et al., 2021; Pan et al., 2019). Given the ratchet-like nature of sex chromosome degeneration, this may reflect differences in the rates of sex chromosome degeneration, but we currently lack the dataset to assess this. To solve this question, we need accurate estimates of the age of sex chromosomes (e.g., from phylogenies and neutral sequence divergence (dS)) along with accurate measures of their degeneration (e.g., gene loss, dNdS, repeat accumulation) from multiple species within multiple distant clades. Indeed, such a task is a prime candidate for the new Tree of Sex database and consortium.

Another mystery relating to sex determination system transitions is their highly heterogeneous rates among taxa. For example, they are very rare in some lineages within insects (Toups & Vicoso, 2023), bryophytes (Carey et al., 2021), cephalopods (Coffing et al., 2024), sturgeons (Kuhl et al., 2021), birds (Fridolfsson et al., 1998) and many other amniote lineages (Kratochvíl et al., 2021a), but very frequent in geckos (Gamble et al., 2015), true frogs (Jeffries et al., 2018), medaka fishes (Ansai et al., 2022; Myosho et al., 2015), and cichlids (El Taher et al., 2021; Feller et al., 2021). Why some sex chromosome systems are so stable, while others transition often, remains an important but unresolved question. Comparative analyses are essential for identifying, dating, and placing transitions on a phylogeny. The Tree of Sex will allow us to do this on an unprecedented scale, and by relating transitions to other life-history traits, we hope to better understand their evolutionary drivers.

### Evolutionary and ecological consequences of reproductive strategies

The reproductive strategy of a given taxon affects its ecology and evolution. For instance, dispersal to, and establishment in a new geographic region can be easier for self-compatible hermaphroditic or asexual species, especially in geographic regions where pollinators or mates are rare (Baker, 1955). This theory has received support in many hermaphroditic plant systems (Grossenbacher et al., 2015; Hargreaves & Eckert, 2014; Pannell et al., 2015; Razanajatovo et al., 2016). Similarly, in animals, simultaneous hermaphroditism can increase fertilization success in unpredictable, unstable, or extreme environments where mates are at low density (Benvenuto & Weeks, 2020), as for parasitic species, and gynogenesis using sperm from other species can result in increased invasion success where conspecific mates are not present (Fuad et al., 2021). For example, parthenogenesis has been linked to range expansion in stick insects (Morgan-Richards et al., 2010). Extending this test of range size to other modes of reproduction would give a more holistic picture of how colonization and establishment can be impacted by reproductive strategy (Tilquin & Kokko, 2016). This could further be used to help predict the potential invasiveness or pathogenicity of species, or their potential for range shifts in response to climate change or other anthropogenic impacts.

Reproductive strategies also influence the ability of taxa to adapt and thus their resilience to biotic or abiotic environmental change. The efficacy of selection is impacted by random associations that occur between alleles that increase fitness and those that decrease fitness limiting both adaptation and the elimination of deleterious mutations (reviewed in Otto, 2021). This process is known as Hill–Robertson effect (Hill & Robertson, 1966) or “selective interference.” Sexual reproduction with recombination can break down these genetic associations and generate variation upon which selection can act. However, some reproductive strategies, such as self-fertilization, limit the effective rate of recombination and therefore exacerbate selective interference (Hartfield, 2016; Hartfield et al., 2017). Similar consequences are predicted in parthenogenetic species, though the exact dynamics depend on the cellular mechanism of parthenogenesis (Engelstädter, 2017). Furthermore, the origin of parthenogenetic species is another factor affecting the levels of selective interference. In addition, the efficacy of selection can be reduced when new parthenogenetic or selfing species form, if they arise spontaneously with little genetic diversity (Jaron et al., 2022).

In the absence of sexual reproduction and effective recombination in a diploid species, homologous haplotypes will independently accumulate mutations resulting in extreme Allelic Sequence Divergence (White, 1945), often also referred to as the “Meselson effect” (Birky, 1996). Consequently, corresponding haplotypes in different individuals and/or populations will be more closely related than two homologous haplotypes within an individual. Despite the elegance of this predicted effect and a large number of parthenogenetic species tested, support is limited (Brandt et al., 2021; Öztoprak et al., 2023; Weir et al., 2016) and evidence in several systems has later been reinterpreted (Freitas et al., 2023; Jaron et al., 2022; Mark Welch et al., 2008; Schwander et al., 2011). Hybridization of distantly related species can generate new parthenogenetic lineages that also show a similar genomic pattern to the Meselson effect (Jaron et al., 2021). Some parthenogenetic species occasionally lose heterozygosity by mitotic recombination between homologous parental chromosomes (Janko et al., 2021), which potentially contributes to mitigation of the accumulation of deleterious mutations due to selective interference (Kočí et al., 2020). In general, the consequences of parthenogenesis have proven less apparent compared to early predictions (Normark, 2003) and are often masked by lineage-specific effects (Jaron et al., 2021). It is of great interest to separate lineage-specific effects and the consequences of the individual reproductive strategies. Only then can we identify the underlying causes of the observed phenomena and finally get closer to answering the question “why sex?.”

In the face of escalating environmental change, determining how different reproductive strategies interact with the environment to influence population resilience is also key for conservation biology. Of the environmental stimuli that can influence organisms, temperature can affect sex determination in many vertebrates (Kitano et al., 2024; Nemesházi & Bókony, 2022; Valenzuela & Lance, 2004). The fast pace of global warming threatens temperature-sex determined vertebrates by disrupting sex ratios and other traits (Honeycutt et al., 2019; Jensen et al., 2018; Lockley & Eizaguirre, 2021; Valenzuela et al., 2019). Similar climate-driven sex-ratio distortions are expected in GSD species that are prone to thermal sex reversal, with complex consequences for population dynamics and microevolution (Bókony et al., 2017; Nemesházi et al., 2021; Schwanz et al., 2020; Whiteley et al., 2018). Further, population dynamics of species with multifactorial sex determination such as GSD houseflies or TSD/GSD silverside fish, where the distribution of sex determination variants is temperature-dependent and geographically clinal (Duffy et al., 2015; Feldmeyer et al., 2008; Foy et al., 2024), will also be affected by climate change. Aside from temperature, sex determination mechanisms may also be disrupted by pollutants that act as endocrine disruptors that directly affect sexual development or induce stress that renders individuals susceptible to thermal insults (Mizoguchi & Valenzuela, 2016). Thus, anthropogenic disruption of the chemical (pollution) and thermal environment (globally or locally, e.g., the urban heat island effect) may have a combined influence on sex determination. For instance, elevated sex-reversal frequency has been observed in agile frogs (*Rana dalmatina*) in areas where anthropogenic land use (i.e., agriculture and urbanization) is high (Nemesházi et al., 2020). By centralizing data for environmental effects on sex determination mechanisms, the Tree of Sex database will allow a comprehensive overview of species that are more vulnerable to unavoidable environmental changes, which could, in turn, support conservation actions.

At a macroevolutionary scale, reproductive strategies play an important role in shaping species diversity. For instance, in the plant family Solanaceae, obligate outcrossing (self-incompatibility) is associated with higher net diversification rates than in species capable of self-fertilization (Goldberg et al., 2010). Even though self-compatible Solanaceae species exhibit a higher speciation rate than obligate outcrossing species, this rate is exceeded by the much higher extinction rate of self-compatible plants. This pattern agrees with theoretical expectations, showing that species barriers accumulate faster in selfing than outcrossing species under most scenarios of speciation (Marie-Orleach et al., 2022). However, the relative importance of genetic (selective interference) and demographic factors in driving higher rates of extinction in self-compatible species remains unclear. Species with separate sexes can exhibit conflicts for traits that have different optima between sexes, leading to an evolutionary arms race that can ultimately contribute to reproductive isolation. A comparative analysis of hybridizing plants supports this idea, with strictly dioecious species pairs exhibiting an excess of species barriers compared to species with other reproductive strategies (Pickup et al., 2019). Sex chromosomes are also expected to facilitate reproductive isolation, as evidenced by the two rules of speciation, namely Haldane’s rule (Haldane, 1922) and the large-X effect (Charlesworth et al., 1987; Coyne, 1985, 2018; Masly & Presgraves, 2007; Turelli & Orr, 1995; Turner & Harr, 2014). These rules have been widely validated using empirical data (reviewed in Beukeboom & Perrin, 2014). Furthermore, hemizygosity of sex chromosomes appears to be a key feature in explaining the disproportionate role of sex chromosomes in speciation (Fraïsse & Sachdeva, 2021; Lima, 2014); however, more work is required to understand the underlying mechanisms. It is also not known what impact sex chromosome turnovers have on reproductive barriers, though many potential mechanisms exist for them to do so. Using the Tree of Sex to relate these factors to rates of branch splitting on a robust phylogeny could lend valuable insights into the role of reproductive strategies in diversification.

Finally, the fundamental transition between isogamy and anisogamy is likely to have had dramatic consequences for sexual trait evolution. It is proposed to have caused stronger sexual selection in males leading to female-biased parental care and male-biased sexual dimorphism (Janicke et al., 2016). Mating systems also influence the costs and benefits of parental care for both sexes. For example, theoretical models predict that males increase the level of care they provide to their offspring when the certainty of their paternity increases (Westneat & Craig Sargent, 1996). However, the empirical evidence for a positive relationship between certainty of paternity and paternal care is mixed (Alonzo, 2010), which calls for more theoretical work and standardized empirical approaches.

In summary, the centralization and integration of reproductive knowledge in the Tree of Sex will allow researchers to address many fundamental questions related to the evolution of reproductive strategies. However, there are many more questions that could be addressed than those listed above. As the database will be a long-standing public resource, our hope is that the wider community will eventually use it to address questions that we have not yet conceived.

## The Tree of Sex rebooted

This new iteration of the Tree of Sex was established in June 2023, almost a decade after the first Tree of Sex project ended. Motivated by the questions and challenges above, we have two major goals: (1) to ***centralize and integrate*** published knowledge related to reproductive biology in a single public database and (2) to ***provide a framework for ethical research and collaboration*** in which to use the database to address scientific questions. Below we outline our plans, current progress and the challenges we foresee for the Tree of Sex consortium and for the database implementation.

### The consortium

**Figure 3.**
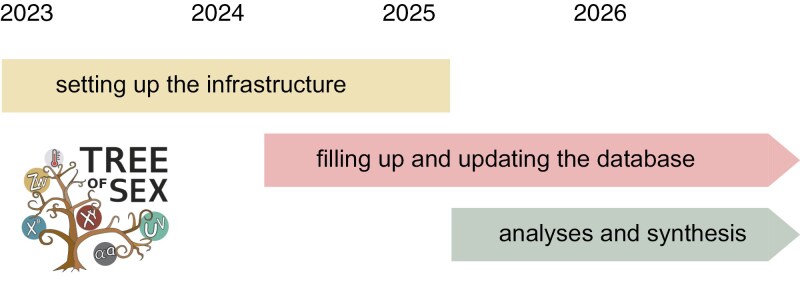
Time plan for the three phases of the Tree of Sex consortium. We are currently advancing in setting up the infrastructure to manage the consortium and create the database. The next phase, starting this year, will be to fill the database with records and continue to keep the database updated. Once the database contains an adequate number of records, we will start analyzing the data by organized topic research teams.

At the time of writing (April 2025), the consortium has approx. 200 members, from 130 institutions across 35 countries. The Tree of Sex consortium is a global community of scientists with the aim of building a collective knowledge base in the field of reproductive biology of eukaryotes. We are currently in the process of establishing the foundations for a long-term, self-sustaining initiative (Figure 3). To accomplish this task, the consortium is managed by a general committee and six “operations teams,” each responsible for specific demands of the consortium (Table 2). These demands are currently focused on setting up the infrastructure necessary for the development of the database in a sustainable and equitable manner.

**Table 2.** Operations teams currently active in the ToS Consortium.

Operations team	Deliverables
Database integration	- listing and describing reproductively relevant databases - facilitating their integration within Tree the Sex Database
Ethics and inclusivity	- guiding and formulating shared values of the consortium towards building a diverse community of scholars and approaching the topics of sex with awareness and sensitivity to its potential societal impact - supporting teams with the work of building the database and challenging decisions that may arise with our values of justice, equity, and inclusion
Funding	- monitoring research funding landscape for opportunities for the consortium and consortium members - supporting individual grant applications of consortium members
Literature search	- generating a list of relevant literature to be processed for the database - exploring opportunities of automated literature processing
Ontology	- create and maintain the Tree of Sex Ontology
Outreach and science communication	- internal communications of the consortium - managing social media and outreach events

Given the volume of literature, numerous subdisciplines and data types, and the great taxonomic breadth we aim to capture, we necessarily depend on the engagement of many experts. The Tree of Sex project is thus, by necessity, a community effort. The consortium is open, and we invite anyone and everyone interested in reproductive biology to join us via the registration form at the Tree of Sex webpage (treeofsex.org). We welcome and encourage researchers from all career levels, nationalities, cultures, genders, and sexual orientations, especially those which have traditionally been minoritized in science.

#### Consortium challenges

Establishing a new consortium comes with several challenges, many of which are shared across projects. Here we will discuss two challenges that specifically concern the Tree of Sex. Firstly, reproduction is an extremely complex biological process but one that is always viewed through a lens of human bias; see de Vries and Lehtonen (2023) and references therein. Our aim within the Tree of Sex is to capture this complexity in a way that is as objective as possible. But how do we accomplish this when recognizing one’s own biases, or those of one’s group, is extremely difficult? We believe that the Tree of Sex consortium *must* equitably include a diversity of people from the start, so that they can co-determine its scope, and recognize each other’s biases. To help accomplish this, the consortium includes a team dedicated to Ethics and Inclusivity (Table 2) whose specific goals centre around building a diverse and inclusive community of scholars. So far, the Ethics and Inclusivity team have laid the foundation for an inclusive and supportive environment for its members through the creation of a code of conduct for members of the consortium and attendees of networking events as well as an ethical vision to be used as a guide for ethical behaviour across all consortium activities. The team is currently working on establishing standard protocols for accreditation of work and systems to initiate discrete projects and allocate leadership and authorship opportunities in an equitable way. The future plans for this team include the tracking of recruitment, retainment, and advancement of consortium members from different backgrounds in order to monitor equitable inclusion and barriers to participation. We also envisage this team collaborating with others in the consortium to organize activities that mitigate inequity among members. Such activities may include, for example, bursaries for travel and childcare, mentorship programmes for students and members of minority groups within the consortium, or facilitating remote access to computing resources required to work with the ever-increasing amounts of genomic data that is relevant to much of the research on reproduction. Global inclusivity is a major outstanding issue in science, and we are committed to working towards an equitable consortium, yet we recognize the challenge in doing this effectively and we will continuously update our activities to do so.

The second challenge is concerning the socially charged nature of the topic of sex. Indeed, terms like sex, sexuality, and gender have a long history of critique from the feminist and LGBTQIA+ groups for their use to marginalize members of these communities (Aghi et al., 2024; Fausto-Sterling, 2008; Richardson, 2013). While the scientific work of the consortium will primarily focus on biological systems that have very little to do with the human-centric sociocultural context of sex, arguments using natural systems are frequently featured in public discussions. Therefore, we must put measures in place to prevent misuse of the collected data in pseudoscience and bigoted narratives. The exact steps we take to do this are a matter of ongoing debate within the consortium, and we welcome input from all members of the scientific community. Interpretations of sex or sexuality can vary widely even among biologists, and we are thus working hard to construct a consortium and an environment where multiple interpretations of sex and related topics can be integrated. We also hope to collaborate with social scientists to discuss how our understanding of reproduction from nature feeds into human societies. And lastly, we are committed to tracking the press arising from the outputs of the consortium, and, where needed, writing public press pieces to counter any erroneous arguments directly based on our work.

### The database

The new Tree of Sex database will store the data at the level of observation (rather than species). This will allow for multiple observations of the same feature (e.g., chromosome numbers) for each species and will enable us to capture conflicting data within the literature regardless of whether they reflect erroneous results or biological polymorphism (e.g., in cases where individuals or populations differ in a reproduction-related trait). The database interface will allow users to aggregate data in multiple ways, including by taxon. For each piece of information stored in the database, the taxon will be recorded in the form of a taxon ID (TaxID) maintained within the International Nucleotide Sequence Database Collaboration. TaxIDs are unique and permanent identifiers for species that aggregate synonyms as well as accommodate species name changes. New identifiers will be requested following the guidelines of NCBI together with the Biodiversity Genomics community (Blaxter et al., 2024b). Each submitted observation will require a reference to its primary source (e.g., scientific article), with multiple submissions from the same reference possible. Finally, each record will be associated with the recorder via their ORCID (required for data submission), and the date of the data entry. These metadata ensure traceability of information and enable crediting active members of the community.

#### Database challenges

The scope and scale of this new version of the Tree of Sex database present several challenges. First, the volume of research on reproduction is vast. The literature search performed to create Figure 1, though based on a limited search prompt, resulted in over 85,000 publications, which is likely the tip of the iceberg for information relevant to the database. Thus, while many papers may be redundant (e.g., thousands of papers on humans and mice), the relevant literature is enormous and growing at a cumulative annual rate of over 4% (Figure 1). Synthesizing this vast and growing literature is a daunting task, even with a large group of volunteer researchers. However, preliminary tests show that use of Large Language Models (LLMs) could greatly speed up this process. LLM tools can be used to interrogate publication PDFs and, with well-constructed prompts, extract specific pieces of information. Our current plans involve integrating these tools in a data extraction pipeline, whereby LLMs produce targeted information summaries of each paper, which are formatted to allow for automatic import into the database if deemed accurate. Experts will be tasked with developing these pipelines and checking summaries against the original paper. The final goal is to construct a hybrid AI-human pipeline in which (ideally) most papers can be automatically imported via the LLM-produced summary, with a proportion (e.g., 10%) being checked by human experts to ground truth their content and estimate error rates. All unchecked database entries will be flagged as such, allowing users to decide how much to trust each data point that they extract, and the database user interface will allow for users to validate unchecked entries if and when they are able, facilitating the continuous increase in proportion of the database that is human validated. In parallel we will periodically implement semi-automated checks of the database contents using biological principles, either via automated scripts or again using AI. For instance, if a single ZW system is found in well-sampled clade that otherwise contains a conserved XY system with a single origin, it could be flagged for manual checking. This is one simple example of dozens of checks that could be implemented to reduce error rates in the database if needed.

The second challenge will be to integrate the information from the literature. This information will span multiple data types, a vast taxonomy, and come from sources spanning over a century. Encoding this information in a database in such a way that is consistent and usable will represent one of the largest challenges in the project. To achieve this, we are developing the Tree of Sex Ontology (TOSO https://github.com/Tree-of-Sex/ToS-Ontology), which will capture the hierarchy of terms within the database and their logical relationships. Using an ontology allows us to integrate information via a published and versioned logic, which will facilitate both data entry and interpretation. For example, consider a scenario in which a researcher wants to enter an observation of automixis for a given taxon. Automixis is a mode of parthenogenesis, thus the logic of TOSO will allow for automatic entry of “parthenogenesis” for reproductive strategy. Using an ontology also allows for logical testing of the consistency of the submitted data as well as identification of possible discrepancies between studies. Finally, the ontology has the capacity to record synonyms and give them context, facilitating the resolution of conflicting or overlapping naming conventions between research disciplines and taxonomic specialities. For example, in the plant literature “apomixis” refers to asexuality via unfertilized seeds, whereas, in the animal literature, “apomixis” refers to a type of asexuality without meiosis and without recombination (mixing) between haplotypes (mitotic asexuality) (Neiman et al., 2014; Van Dijk, 2009). The ontology allows contextual definitions of these terms that can be used in parallel, and users will be able to both submit data and query the database without enforcing any of their individual community’s standards on others. TOSO will therefore be the tool by which we integrate all information within the Tree of Sex and will help us unify this currently disparate field.

## Outlook

Compiling all knowledge relevant to the diversity of reproductive strategies in eukaryotes is ambitious but promises to advance the field of evolutionary biology. The integration of information across the taxa and topics will increase understanding of the patterning of reproductive strategies across the eukaryote phylogeny, identify gaps towards which we can direct future research, and facilitate large-scale comparative analyses to test fundamental evolutionary questions. However, this task will require a large community effort thus we warmly welcome researchers from all locations and backgrounds who are directly or indirectly interested in reproduction to join the Tree of Sex consortium (treeofsex.org).

## Glossary

Below are definitions of some important terms used in this article. Where possible we have adopted current definitions from existing biological ontologies (see term IDs). In some cases, we have slightly modified definitions to match our use of the term (see “Adapted from”). For terms lacking a definition in existing ontologies, or for which the existing definition(s) is inadequate, we have adopted new definitions based on our own current understanding.

We acknowledge that our interpretation of some of the terms below may reflect some of our biases as researchers, and that some definitions may not include the full diversity of cases that exist in nature. Thus, the definitions below are presented to clarify our use of terms in this manuscript, and not as the “only” or “correct” definition for a given term.

We also note that definitions of terms may change from those presented below as our biological understanding expands. These changes will be reflected in the ontology generated by the consortium as part of the ongoing work that the Tree of Sex is conducting. This ontology, will include precise and inclusive definitions connected by logical relationships between terms, that will eventually be used for the database.

**Table:** 

**Anisogamy**	A characteristic of sexually reproducing organisms whereby the different gamete types produced by a species differ substantially in size or form.
**Arrhenotoky**	A sex determination strategy in which males develop from unfertilized eggs and are haploid, and females develop from fertilized eggs and are diploid. From GSSO:000121
**Asexual reproduction**	A type of reproduction in which new individuals are produced from a single organism, either from an unfertilized egg or from a single cell or group of cells. From GO:0019954
**Automixis**	In the context of fungal reproduction, the fusing gametes might be from the same meiotic tetrad. In plants and animals, it refers to a form of parthenogenesis, where the progeny is not genetically identical to the parent.
**Dioecy** **(synonym: Gonochorism)**	Sexual strategy in which anisogamous (macro or micro) gametes are produced in separate individuals. Dioecy is used in plants while gonochorism typically refers to animals.
**Dioicy**	When sex is determined in the haploid stage, the occurrence of gametophytes that produce only one type of gametes; separate sexes.
**Environmental sex determination (ESD)**	The determination of sex by a non-genetic cue, such as temperature, nutrient availability, or social context. Adapted from GSSO:000107
**Female**	In anisogamous organisms, a category of individual or organ (e.g., flower) sex which produces macrogametes (ova). Note that this simplified categorization may not be reflective of existing phenotypic diversity as it ignores the complex and often overlapping combination of other traits associated with sex.
**Gametes**	A mature sexual reproductive cell often (but not always) having a single set of chromosomes (haploid). Adapted from CL:0000300
**Gametolog**	One of a homologous set of genes in the non-recombining region on the sex chromosomes. Can also be used to refer to the individual sex chromosomes themselves (e.g., the X and Y chromosomes are the two gametologs of an XY system).
**Gametophyte**	The multicellular haploid stage of the life cycle of a (sexual) plant species in which gametes are produced.
**Genotypic sex determination (GSD)**	Determination of sex by genotypic constitution, namely by chromosomal or genetic factors consistently associated with each sex. Adapted from GSSO:000104
**Gonochorism** **(synonym: Dioecy)**	Sexual strategy in which anisogamous (macro or micro) gametes are produced in separate individuals. Gonochorism typically refers to animals, whereas dioecy is used in plants.
**Haploid**	Chromosomally reduced stage of the life cycle. In some species, the reduced stage is the relatively short phase with gametes directly produced during meiosis; in others (fungi, mosses, ferns) meiosis produce sporophytes that undergo mitotic divisions to form a multicellular body before gametes are produced. Note that haploid is often used to specifically refer to a cell containing a single set of chromosomes (i.e., monoploid), though here we use it only to mean reduced.
**Hermaphroditism**	An umbrella term for a suite of sexual reproductive strategies whereby individuals can produce both macro and microgametes. In plants, hermaphroditism refers specifically to plants with flowers that can produce both male and female gametes, and is distinct from Monoecy (see below). See also “Sequential hermaphroditism” and “Simultaneous hermaphroditism.”
**Heterogametic**	A characteristic of an individual producing gametes that differ in sex chromosome constitution, such as heterogametic males in XX/XY (or females in ZZ/ZW systems) that produce X- or Y-containing sperm (or Z- or W-containing eggs).
**Heteromorphic sex chromosomes**	A sex chromosome system in which the sex chromosomes (e.g., X and Y) are morphologically different from each other when observed via microscopy.
**Homogametic**	A characteristic of an individual producing gametes with identical sex chromosome constitution, such as females in systems (XX/XY) (or males in ZZ/ZW systems), that produce exclusively X-containing eggs (or Z-containing sperm).
**Homomorphic sex chromosomes**	A sex chromosome system in which the sex chromosomes (e.g., X and Y) are morphologically indistinguishable when observed via microscopy.
**Intragametophytic selfing**	A form of self-fertilization between gametes produced within the same gametophyte.
**Isogamy**	A characteristic of sexually reproducing organisms whereby the different gamete types produced by a species do not differ substantially in size or shape.
**Male**	In the context of anisogamy, a category of individual or organ (e.g., flower) sex that produces microgametes (sperm). Note that this simplified categorization may not be reflective of existing phenotypic diversity as it ignores the complex and often overlapping combination of other traits associated with sex.
**Mating type**	A characteristic of gametes or individuals of sexually reproducing (often isogamous) species that controls their compatibility for genetic mixing (fusion or mating, respectively).
**Monoecy**	A reproductive strategy In plants, wherein individual plants possess both male and female flowers. Note that this is distinct from “hermaphroditism.”
**Monoicy**	When sex is determined in the haploid stage, the occurrence of gametophytes that produce both male and female gametes.
**Neo-sex chromosome**	A sex chromosome resulting from the fusion of either gametolog of an existing sex chromosome pair (e.g., X or Y to an autosome, which generates a Neo-X chromosome, or a Neo-Y chromosome).
**Parthenogenesis**	Development of an egg into an embryo without being fertilized. MP:0009443
**Programmed DNA elimination**	A process in which genomic fragments or entire chromosomes are eliminated from somatic cells. GO:0031049
**Pseudoautosomal region**	The region(s) of a sex chromosome which still recombine(s).
**Reproductive strategy**	The set of traits (morphological, physiological, behavioral, etc.), that help an organism reproduce.
**Self-compatibility**	The opposite of “Self incompatibility,” i.e., gametes of different types from the same organism are able to fuse, resulting in reproduction.
**Self-incompatibility**	A self-recognition mechanism reducing self-fertilization and inbreeding. Adapted from TO:0000310
**Sequential hermaphroditism**	Sexual reproductive system of some taxa whereby individuals switch between producing either macro or microgametes at some point in their lifetime
**Sex chromosome**	A chromosome harbouring a locus involved in sex determination. Sex linkage within a sex chromosome may be restricted to this locus, or extend, via recombination suppression, across varying proportions of its length. Adapted from GO:0000803.
**Sex**	A category of individual, tissue, genotype, or gamete in anisogamous taxa that undergo sexual reproduction. Applied based on primary or secondary sexual characteristics. Common categories for sexes include female and male, although these generally represent a simplification of phenotypic diversity and continuous variation. Adapted from PATO:0000047. Sometimes akin to mating type in isogamous organisms.
**Sexual reproduction**	A type of reproduction in anisogamous or isogamous eukaryotes that combines the genetic material of two gametes, from two individuals or from a single individual. Adapted from GO:0019953.
**Sexual system**	The pattern of sex allocation and/or mating behaviour across individuals in a sexually reproducing species (e.g., gonochorism, hermaphroditism, mixed sexual systems). Adapted from GSSO:011864
**Simultaneous hermaphrodites**	Sexual reproductive system of some taxa whereby an individual is capable of producing both macro and microgametes at the same time.
**Syngamy**	The union of gametes of types to form a zygote during sexual reproduction. Syngamy involves the fusion of cytoplasm (plasmogamy) and nuclei (karyogamy). Some species live a substantial proportion of their lives in between these two stages (fungi). From GO:0009566

## Data Availability

No new data are presented in this manuscript. Details of the analysis in Figure 1 can be found in the legend.
